# Path Planning and Motion Control of Robot Dog Through Rough Terrain Based on Vision Navigation

**DOI:** 10.3390/s24227306

**Published:** 2024-11-15

**Authors:** Tianxiang Chen, Yipeng Huangfu, Sutthiphong Srigrarom, Boo Cheong Khoo

**Affiliations:** Mechanical Engineering, National University of Singapore, Singapore 117576, Singapore

**Keywords:** quadruped robot dog, model predictive control, whole-body control, multi-level trajectory planner, path planning, depth vision-based navigation, map generation, rough terrain, SLAM

## Abstract

This article delineates the enhancement of an autonomous navigation and obstacle avoidance system for a quadruped robot dog. Part one of this paper presents the integration of a sophisticated multi-level dynamic control framework, utilizing Model Predictive Control (MPC) and Whole-Body Control (WBC) from MIT Cheetah. The system employs an Intel RealSense D435i depth camera for depth vision-based navigation, which enables high-fidelity 3D environmental mapping and real-time path planning. A significant innovation is the customization of the EGO-Planner to optimize trajectory planning in dynamically changing terrains, coupled with the implementation of a multi-body dynamics model that significantly improves the robot’s stability and maneuverability across various surfaces. The experimental results show that the RGB-D system exhibits superior velocity stability and trajectory accuracy to the SLAM system, with a 20% reduction in the cumulative velocity error and a 10% improvement in path tracking precision. The experimental results also show that the RGB-D system achieves smoother navigation, requiring 15% fewer iterations for path planning, and a 30% faster success rate recovery in challenging environments. The successful application of these technologies in simulated urban disaster scenarios suggests promising future applications in emergency response and complex urban environments. Part two of this paper presents the development of a robust path planning algorithm for a robot dog on a rough terrain based on attached binocular vision navigation. We use a commercial-of-the-shelf (COTS) robot dog. An optical CCD binocular vision dynamic tracking system is used to provide environment information. Likewise, the pose and posture of the robot dog are obtained from the robot’s own sensors, and a kinematics model is established. Then, a binocular vision tracking method is developed to determine the optimal path, provide a proposal (commands to actuators) of the position and posture of the bionic robot, and achieve stable motion on tough terrains. The terrain is assumed to be a gentle uneven terrain to begin with and subsequently proceeds to a more rough surface. This work consists of four steps: (1) pose and position data are acquired from the robot dog’s own inertial sensors, (2) terrain and environment information is input from onboard cameras, (3) information is fused (integrated), and (4) path planning and motion control proposals are made. Ultimately, this work provides a robust framework for future developments in the vision-based navigation and control of quadruped robots, offering potential solutions for navigating complex and dynamic terrains.

## 1. Introduction

Quadruped robots have gained significant attention in recent years due to their adaptability and scalability in various environments, such as those in industrial, military, and disaster relief operations. Unlike wheeled robots, quadruped robots can easily navigate stairs and low obstacles, making them ideal for rugged terrains and densely forested areas, where wheeled robots struggle [1]. This ability to traverse complex terrains has propelled quadruped robots to the forefront of modern robotics research. Researchers and developers are currently focusing on enhancing their autonomy, load-bearing capacity, and gait efficiency by integrating advanced control algorithms and artificial intelligence, enabling these robots to execute complex tasks with greater efficiency and flexibility [2].

Despite these advancements, the current navigation and control capabilities of quadruped robots still require significant improvements, especially in complex and dynamic environments. Most traditional navigation systems rely on static, predefined paths, lacking adaptability to unforeseen obstacles and dynamic changes in terrain [3]. For instance, path planning algorithms that are effective in static environments face significant limitations in real-time adaptability and computational efficiency when applied to quadruped robots navigating complex terrains [4]. Additionally, maintaining stability and smooth operation at high speeds or during dynamic maneuvers remains a challenge [5]. For example, the MIT Cheetah robot, known for its speed and agility, struggles to maintain balance and stability when transitioning between different terrains or executing rapid maneuvers [6]. This instability stems from the lack of integration between high-frequency sensor feedback and low-latency control algorithms necessary for real-time adjustments [7]. Moreover, control strategies like WBC and MPC, while robust, are computationally intensive. They require precise modeling of the robot’s kinematics and dynamics, which becomes increasingly complex at higher speeds or when encountering unexpected changes in terrain [8]. The computational demand for real-time optimization often exceeds the capabilities of onboard processors, leading to delayed responses that can compromise stability and increase the risk of falls or collisions [9]. Recent studies suggest that integrating reinforcement learning techniques with traditional control strategies could enhance real-time decision-making capabilities, but this approach has yet to be fully realized in practical applications [10].

Alongside motion control, navigation and trajectory planning present another set of critical challenges. Traditional methods, such as grid-based and sampling-based planning algorithms, often fail to adapt efficiently to dynamic environments due to their high computational costs and suboptimal path generation [11]. These methods typically rely on static environment models and predefined paths, which do not account for dynamic obstacles or varying terrain conditions. As a result, the generated trajectories may not be feasible or safe in real-time applications, especially in environments that require frequent adjustments and recalculations of optimal paths [12]. Furthermore, current quadruped robot systems lack a comprehensive framework that seamlessly integrates navigation, trajectory planning, and motion control. This disjointed nature often leads to inefficiencies and conflicts in decision-making processes [13]. For example, an optimal trajectory generated by the navigation system may be impractical for the motion control system to execute due to its own constraints and limitations, resulting in suboptimal performance, increased energy consumption, and reduced operational safety [14].

To address these challenges, this paper proposes an integrated system that combines robust navigation algorithms, efficient trajectory planning methods, and advanced motion control strategies. The integration of these components allows for real-time adaptability and stability, even in dynamic and unpredictable environments [15]. By leveraging multi-sensor fusion and machine learning techniques, the proposed system optimizes the entire navigation and control process for quadruped robots, providing a more reliable and efficient solution than current methods. The primary objectives of this research are to develop a robust path planning and dynamic control framework that integrates vision-based navigation with advanced motion control techniques for quadruped robots in complex terrains and to validate the proposed framework through extensive simulations and real-world experiments [16]. Key contributions include the development of a customized 2D EGO-Planner for optimized trajectory planning in dynamically changing terrains, the integration of MPC and WBC to improve stability and maneuverability, and the implementation of a multi-sensor fusion system that combines data from depth cameras and inertial measurement units (IMUs) to enhance environmental perception and navigation accuracy.

The remainder of this paper is organized as follows: Section 2 reviews the related work on quadruped robot navigation and control systems. Section 3 and Section 4 detail the proposed methodology, including system architecture, trajectory planning framework, and control algorithms. Section 5 presents the experimental setup and results, highlighting the effectiveness of the proposed approach. Section 6 discusses the findings and summarizes the key contributions, potential improvements, and future work.

### Objectives

This research focuses on enhancing quadruped robot navigation and motion control in complex environments by integrating advanced algorithms and control systems. The primary objective is to adapt the EGO-Planner for 2D navigation, optimizing path planning by limiting unnecessary Z-axis movement. Additionally, this work aims to implement a combined MPC and WBC framework to improve real-time motion planning and ensure robust control in dynamic environments. Furthermore, the research compares different sensor fusion systems, highlighting the advantages of vision-based navigation over LiDAR in trajectory planning and localization, particularly in terms of reduced data requirements and cost-efficiency.

## 2. Literature Review

### 2.1. Robot Dog Platform

Recent advancements in quadruped robots, or robotic dogs, have demonstrated significant progress in their capabilities, particularly in motion control and terrain adaptability. Notable examples include Boston Dynamics’ Spot, MIT’s Cheetah, and ETH Zurich’s ANYmal. These platforms integrate dynamic balancing algorithms and real-time feedback control systems, enabling robust navigation in complex environments. For instance, Spot utilizes stereo vision, gyros, and accelerometers to process feedback for real-time control [17], while MIT’s Cheetah leverages precise force control and high-frequency leg dynamics to maintain stability at high speeds [18]. ETH Zurich’s ANYmal, by contrast, focuses on using machine learning to dynamically plan movements and adapt to varying terrains through real-time sensor fusion, including LIDAR and vision systems [19].

The research community’s advancements are also reflected in commercial models. Unitree Robotics’ A1 and DeepRobotics’ Jueying Lite have expanded accessibility by providing open interfaces and modular designs that support the integration of custom control algorithms. Unitree’s A1, known for its robust mobility and adaptability, excels in industrial and public safety applications by autonomously optimizing its walking strategies [20]. Meanwhile, Jueying Lite prioritizes cost-effectiveness and flexibility, making it a popular platform for consumer and research applications [21].

### 2.2. Trajectory Planning Algorithms

Trajectory planning plays a pivotal role in robot navigation, ensuring that the optimal path from a starting point to a goal is found while accounting for environmental constraints and dynamic changes. This process is typically divided into two key components: global and local planning.

#### 2.2.1. Existing Path and Local Planning Approaches

In robot navigation, global path planning and local trajectory planning are two essential components. Global path planning aims to calculate a feasible path from a starting point to a goal, typically using static or existing maps. Traditional algorithms, such as A* and its variant D* Lite, are widely used for this purpose. A* is efficient in static environments for finding the shortest path; however, in dynamic environments with moving obstacles, it requires frequent path recomputation, which can significantly increase computational cost and reduce real-time performance [22]. Dynamic A* (D*) improves this by updating only the affected portions of the path in dynamic environments, reducing computational overhead and improving adaptability [23]. Despite these advancements, D* mainly serves as a static path generator and is less effective in scenarios requiring real-time updates during robot motion.

However, local path planning addresses real-time environmental changes, often dealing with unpredictable obstacles or dynamic conditions. The Dynamic Window Approach (DWA) is a common method, optimizing the robot’s velocity and trajectory based on its current state and nearby obstacles. While effective in static environments, the DWA struggles with dynamic obstacles and often gets trapped in local minima [24]. Another approach, the Time Elastic Band (TEB), adjusts trajectory nodes to improve path smoothness and efficiency while considering the robot’s kinematic constraints. Although the TEB enhances path efficiency, it faces computational challenges in highly dynamic environments, limiting its real-time application [25]. These limitations underscore the need for more adaptive local planners in complex and changing environments.

#### 2.2.2. Improvements in Path and Local Trajectory Planning

This study addresses the limitations of existing global and local planning algorithms by utilizing established methods while introducing key improvements. For global path planning, we employ the Dynamic A* (D*) algorithm to generate an initial path. D* serves as the foundation for static obstacle avoidance but remains a preliminary path generator [26]. The global path generated by D* acts as a general guideline for robot navigation without any modifications to the algorithm.

The main contribution of this study lies in the local trajectory planning through the adaptation of the EGO-Planner into a two-dimensional (2D) version, specifically optimized for ground-based quadruped robots. Unlike traditional planners that rely on existing global maps [27], this 2D EGO-Planner dynamically updates the trajectory using real-time sensor feedback, allowing the robot to navigate complex, unknown environments [28].

The global path produced by D* serves as an initial rough input to the 2D EGO-Planner, which not only acts as a dynamic path optimizer but also incorporates kinematic constraints. The final output is a time-parameterized trajectory that includes the robot’s positions, velocities, and accelerations over time. Specifically, at each time step *t*, the planner computes the center of mass (CoM) positions p(t), velocities v(t), and accelerations a(t) as $\left\{p\left(t\right),v\left(t\right),a\left(t\right)\right\},t\in \left[t_{0},t_{f}\right]$. This planner ensures real-time adjustments based on sensory data, improving adaptability and reducing the likelihood of the robot becoming trapped in local minima. A more detailed explanation of this adaptation is provided in Section 4.6.

### 2.3. Control Systems for Quadruped Robots

Control systems for quadruped robots are typically divided into low-level controllers and high-level planners. Low-level controllers handle joint control and stability, while high-level planners generate motion trajectories and adapt to dynamic environments. Coordination between these layers is essential for robust locomotion, especially in unpredictable terrains. Various approaches have been proposed to address challenges in both layers, optimizing performance across different conditions.

#### 2.3.1. Existing Control Strategies and Limitations

Low-level strategies, like PD control, are essential for joint-level motion but are limited in adapting to dynamic and unpredictable environments. Advanced methods, including computed torque control and sliding mode control, have been explored but still struggle with real-time adaptation to complex terrains. High-level methods, such as Zero-Moment Point and inverse kinematics (IK)-based planning, are effective in specific locomotion tasks but often lack the flexibility to adapt to dynamic, unstructured environments due to their reliance on pre-planned motions [29].

To overcome these limitations, MPC has been widely adopted for its adaptability in complex environments. By considering the robot’s current state and dynamic constraints, MPC provides robust motion optimization, while Whole-Body Control (WBC) ensures the seamless execution of planned trajectories. This approach has proven effective in real-world applications, such as MIT’s Cheetah robot [30].

#### 2.3.2. Implementation and Adaptation

Adapting the MPC and WBC combination to our quadruped robot required addressing challenges related to the mechanical structure, including motor specifications and joint torque limits. Additionally, the integration of foot-end force sensors for real-time feedback demanded precise calibration to ensure accurate torque control. We used PD controllers at the low level to execute the high-level commands generated by MPC and WBC, controlling each motor’s torque and ensuring precise joint-level movement. After resolving these hardware challenges, we successfully established a control framework, laying a foundation for dynamic stability and future navigation experiments.

## 3. Dynamic Motion Control

### 3.1. Hardware Overview of the Quadruped Robot

**Figure 1:**
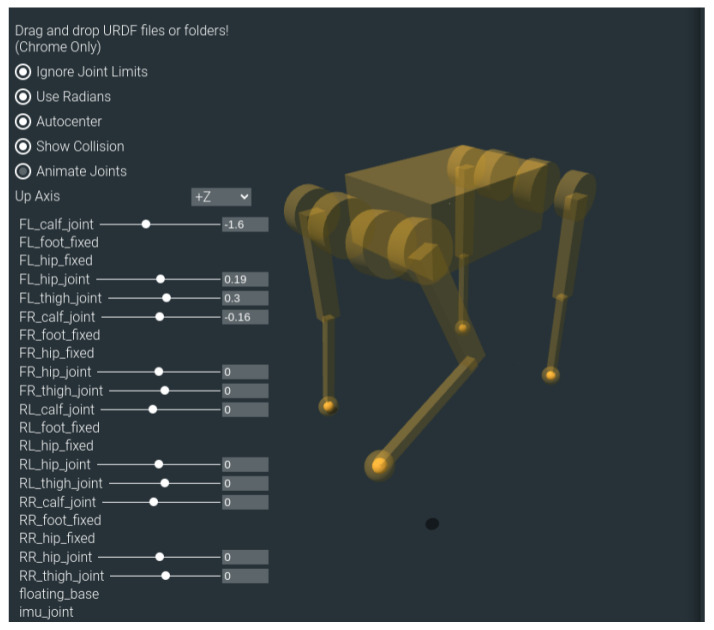
Simplified box model of the Lite3P quadruped robotic dog.

**Figure 2:**
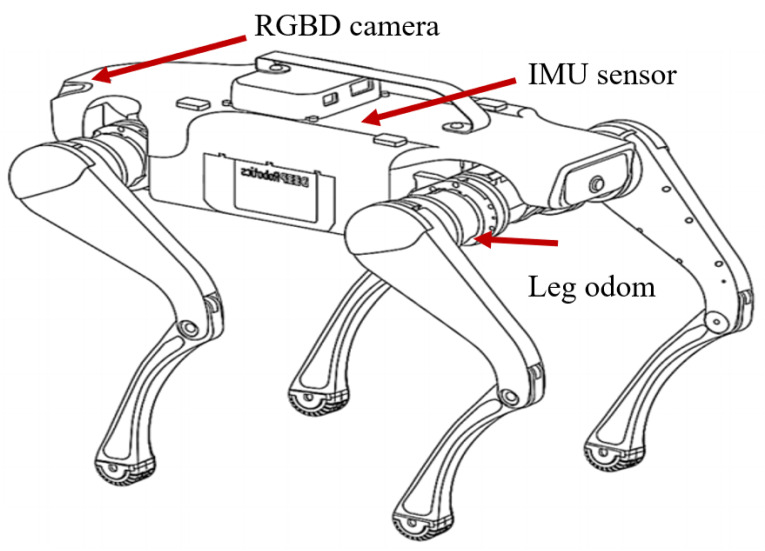
Internal sensor arrangement of the quadruped robotic dog.

In this study, we built a quadruped robotic dog simulation platform based on Gazebo and RViz, and we integrated multiple sensors, including RGB-D cameras, IMUs, and odometers, using an Extended Kalman Filter (EKF) to improve state estimation [31]. The robotic dog used in our simulations is the Lite3P model developed by Unitree Robotics, as shown in Figure 1. This robot features a simplified 12-degree-of-freedom (DOF) structure, with each leg having three joints: a hip, a thigh, and a calf. This mechanical configuration is sufficient to support subsequent experiments. The hardware layout of these sensors is shown in Figure 2. The Intel RealSense D435i depth camera provides high-precision RGB-D data, which are crucial for environmental perception. The IMU, a six-DOF sensor, collects real-time acceleration and angular velocity data, and it is installed in the center of the robot’s body to help estimate its posture during movement. Additionally, foot odometers are installed at the hip joints of each leg, monitoring joint angles, movement speed, and torque, ensuring precise control of the robot’s gait.

### 3.2. Physics Modeling of the Quadruped Robot

To improve the performance of real-time trajectory planning and to enhance the computational efficiency of the control algorithms, we employ a simplified centroidal dynamics model. This approach has been widely utilized in humanoid and mobile robotics, where representing the robot as a point mass at its CoM significantly reduces computational complexity while preserving essential dynamic characteristics [32]. In our two-dimensional EGO-Planner framework, the trajectory planner focuses solely on the robot’s CoM trajectory rather than explicitly planning the motions of the individual legs, following similar strategies applied in biped locomotion planning [33]. The discrete CoM trajectory points generated by the planner are then fed into an MPC and WBC framework. Within this control framework, inverse kinematics plays a critical role in translating the desired CoM trajectory into joint-level actions. Specifically, the WBC component utilizes IK to compute the joint angles required to achieve the target foot-end positions generated by MPC. This process relies on the pseudo-inverse of the Jacobian matrix, ensuring precise control of each leg while satisfying physical constraints such as joint limits and ground contact stability. The centroidal dynamics model, which treats the robot as a single point mass, facilitates the description of the robot’s physical behavior using simplified dynamic equations. This approach provides an efficient framework for later predictive optimization and control.

The equations governing the dynamics of the robot’s CoM and rotational motion are given in Equation (1) and Equation (2), respectively:

(1) $$m\ddot{p}=\sum_{i=1}^{n_{e}}f_{i}-c_{g}$$

(2) $$\frac{d}{dt}\left(I\omega \right)=\sum_{i=1}^{n_{e}}r_{i}\times f_{i}$$

Here, *m* represents the total mass of the robot, $\ddot{p}$ is the acceleration of the CoM, and $f_{i}$ is the ground reaction force acting at leg *i*. The rotational dynamics are described by the angular velocity ω, with the inertia tensor *I* governing the rotational inertia of the robot’s body. The meanings of these parameters follow the standard definitions commonly used in centroidal dynamics models for humanoid and legged robots [32].

In subsequent experiments, the centroidal model calculates the dynamic behavior of the robot’s CoM, providing information on the position, velocity, and acceleration of the CoM for correction by the EKF. When solving joint torques and distributing foot contact forces in the WBC, the floating-base dynamics model and the multi-body dynamics model are employed. The floating-base dynamics model calculates the motion state of the CoM and its relationship with external forces [34], while the multi-body dynamics model describes the relative motion between the robot’s limbs and torso, computing joint torques and force distribution [35]. However, as the focus of this section is on the MPC and WBC control processes, further details of the floating-base dynamics and multi-body dynamics models are omitted. Readers interested in these models can refer to References [34,35].

### 3.3. Dynamic Motion Control Model

As previously mentioned, the motion control of quadruped robots involves complex full-body dynamics and coordination between the limbs. Consequently, motion planning in complex terrains cannot be achieved through simple point-to-point trajectory planning. To address this challenge, we integrated a control strategy from Cheetah with an existing centroidal dynamics model to enable efficient motion control for quadruped robots [30].

**Figure 3:**
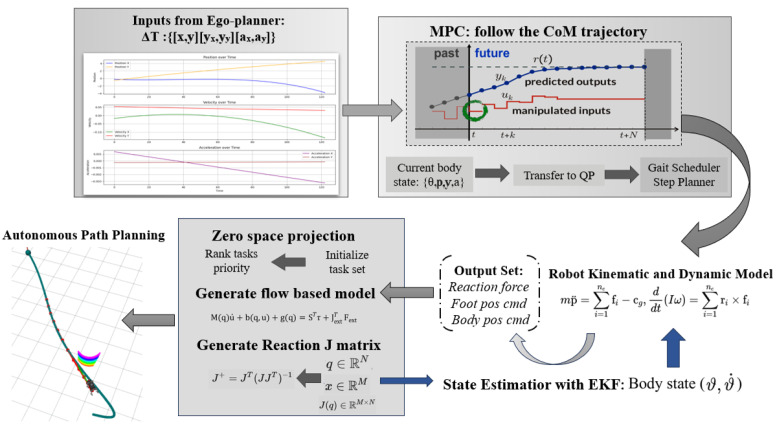
Dynamic control flowchart.

As shown in Figure 3, once the 2D EGO-Planner generates the COM trajectory $\left(\vartheta_{t},{\dot{\vartheta }}_{t}\right)$, it is fed into the MPC module. Using this trajectory and the robot’s dynamic model, the MPC calculates the desired foot positions, centroid orientation, and contact forces at each time step. WBC then integrates these outputs to compute the contact forces and optimal joint torques. The motors execute these control commands, incrementally guiding the robot towards the target posture. During operation, sensors such as the IMU and foot-end odometers provide real-time feedback, while forward kinematics calculates the actual foot positions. An EKF fuses these data with the expected values to estimate the robot’s posture and position. This updated state information is sent to the upper-level planner to adjust the trajectory until the robot reaches the target.

#### 3.3.1. MPC Model

The basic principles of MPC include predictive modeling, receding horizon optimization, and feedback correction. During the predictive modeling phase, MPC predicts future outputs based on current and historical inputs. Receding horizon optimization continuously recalculates the optimal control sequence at each sampling time. Meanwhile, feedback correction adjusts the control inputs by comparing the actual output with the predicted output, ensuring accurate trajectory tracking. By combining the centroidal model mentioned in Section 3.2 and relevant assumptions, we introduce the process of deriving the system’s dynamic model for MPC.

Based on this formulation, the discrete dynamic representation of the system is expressed in Equation (3) as follows:

(3) $$x\left(k+1\right)=A_{k}x\left(k\right)+B_{k}\hat{f}\left(k\right)+\hat{g},$$

where x(k), $A_{k}$, $B_{k}$, $\hat{f}\left(k\right)$, and $\hat{g}$ represent the state vector, system matrices, contact forces, and environmental forces, respectively. These parameters follow the standard definitions widely used in MPC formulations, where x(k) consists of the position, velocity, and orientation of the robot’s CoM; $A_{k}$ and $B_{k}$ represent the system and input matrices; and $\hat{f}\left(k\right)$ includes the contact forces acting on the robot [36].

The state vector x(k) is defined as

(4) $$x={\left[\Theta^{⊤},p^{⊤},\omega^{⊤},p^{⊤}\right]}^{⊤},$$

where Θ represents the orientation of the robot’s base, p represents the position of the robot’s CoM, and ω refers to the angular velocity of the robot’s base.

The quadratic programming (QP) optimization problem for MPC is then posed to minimize the deviation between the predicted state and the reference trajectory while balancing the control effort. The weight matrices Q and R, which control the trade-off between tracking accuracy and energy efficiency, are also standard in MPC formulations [37].

Next, the system matrices $A_{k}$ and $B_{k}$, which describe the time evolution of the CoM and the impact of the contact forces, are defined in Equations (5) and (6) as follows:

(5) $$A=\left[\begin{array}{cccc} 1_{3\times 3} & 0_{3\times 3} & R_{2}\left(\psi_{k}\right)\Delta t & 0_{3\times 3}\\ 0_{3\times 3} & 1_{3\times 3} & 0_{3\times 3} & 1_{3\times 3}\Delta t\\ 0_{3\times 3} & 0_{3\times 3} & 1_{3\times 3} & 0_{3\times 3}\\ 0_{3\times 3} & 0_{3\times 3} & 0_{3\times 3} & 1_{3\times 3} \end{array}\right],B=\left[\begin{array}{ccc} 0_{3\times 3} & \cdots & 0_{3\times 3}\\ 0_{3\times 3} & \cdots & 0_{3\times 3}\\ G^{-1}{\left[r_{1}\right]}_{\times }\Delta t & \cdots & gI^{-1}{\left[r_{n}\right]}_{\times }\Delta t\\ 1_{3\times 3}\Delta t/m & \cdots & 1_{3\times 3}\Delta t/m \end{array}\right].$$

To minimize the deviation between the system’s predicted state and the reference trajectory, the following QP optimization problem is posed in Equation (6):

(6) $$\min_{x,f}\sum_{k=0}^{m}∥x\left(k+1\right)-x^{\mathrm{ref}}{\left(k+1\right)∥}_{Q}+{∥f\left(k\right)∥}_{R}.$$

This objective function seeks to reduce both the deviation from the desired trajectory and the magnitude of the control inputs while also considering energy consumption constraints [37].

**Figure 4:**
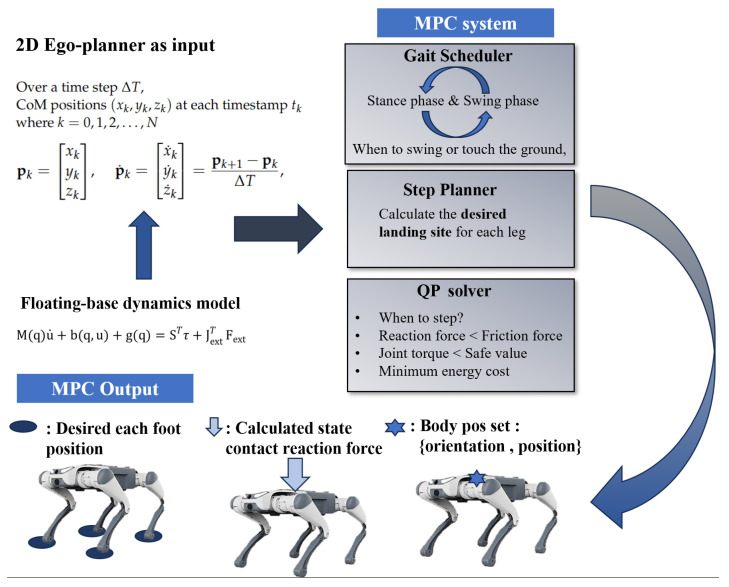
MPC flowchart.

As shown in Figure 4, MPC acts as the upper-level controller, receiving geometric information about CoM from the 2D EGO-Planner, which includes time series data of velocity and position. The CoM position is given by

(7) $$p_{k}=\left[\begin{array}{c} x_{k}\\ y_{k}\\ z_{k} \end{array}\right],\;{\dot{p}}_{k}=\left[\begin{array}{c} {\dot{x}}_{k}\\ {\dot{y}}_{k}\\ {\dot{z}}_{k} \end{array}\right]=\frac{p_{k+1}-p_{k}}{\Delta T}$$

where $p_{k}$ represents the CoM position at time $t_{k}$, and ${\dot{p}}_{k}$ represents the corresponding velocity. Equation (7) gives the expression of the planned CoM position and the CoM velocity.

In the MPC computation process, the floating-base dynamics model is integrated to improve accuracy in deriving the dynamic behavior of the CoM. The use of the floating-base model allows the controller to account for the robot’s degrees of freedom, providing a more accurate representation of CoM attitude changes; the attitude of the CoM is mainly computed by maintaining balance. MPC computes the forces exerted by each leg on the CoM and uses these forces to derive changes in the CoM’s attitude. The CoM acceleration is calculated using the following equation:

(8) $$\ddot{r}=\frac{1}{m}\sum_{i=1}^{4}f_{i}+g$$

where $\ddot{r}$ represents the CoM acceleration, *m* is the robot’s mass, $f_{i}$ represents the contact forces from each leg, and g accounts for gravitational acceleration.

The rotational state of the CoM is described by angular velocity and angular acceleration. By leveraging the relationship between torque and angular acceleration, MPC generates the expected foot-end positions and contact forces for each leg. The relationship between the torque and angular acceleration is given by

(9) $$\tau =I\ddot{\theta }+\omega \times I\omega$$

where τ is the applied torque, *I* is the inertia tensor, $\ddot{\theta }$ is the angular acceleration, and ω is the angular velocity. This enables MPC to optimally track the target trajectory at each time step.

As illustrated in Figure 4, the gait scheduler determines the motion phase of each leg according to a predetermined gait cycle. The step planner then calculates the desired touchdown position for each leg based on the CoM trajectory generated by MPC. Key constraints, such as maintaining ground contact forces within friction limits and avoiding excessive joint torques, are considered to ensure efficient and balanced locomotion. The MPC framework optimizes foot-end positions and contact forces while minimizing energy consumption and torque expenditure, formulated as a QP problem. A detailed discussion of these gait scheduling and step planning processes is beyond the scope of this study and is therefore omitted.

#### 3.3.2. WBC Model

In previous sections, we frequently referenced WBC, an optimization-based strategy for coordinating joint torques and forces in robots. WBC sends optimized commands to a lower-level PD controller, taking into account the dynamic balance of the entire system and the interactions between various tasks, thus ensuring coordinated motion across all joints. In this paper, we adopt a hierarchical control strategy for WBC, where tasks are prioritized by their importance [38]. As shown in Table 1, tasks such as optimizing the contact forces for the supporting legs (highest priority), controlling the base’s position and orientation, and managing the swing leg’s trajectory (lowest priority) are handled accordingly.

**Figure 5:**
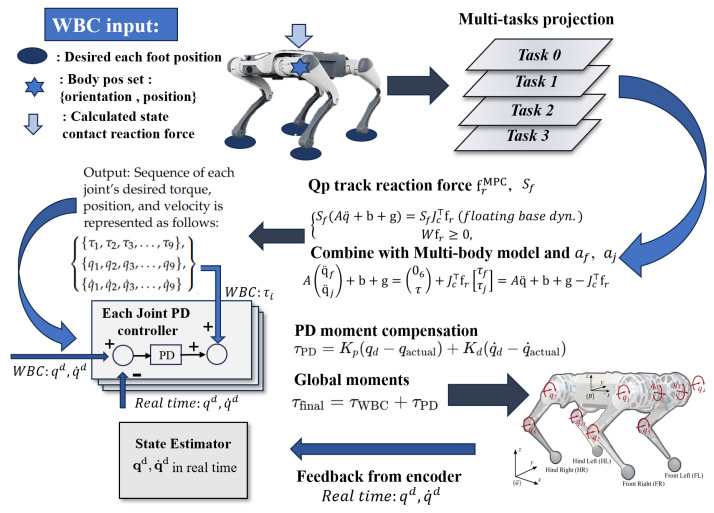
WBC flowchart [30].

This hierarchical control strategy is illustrated in Figure 5. During the execution of WBC, the control algorithm first addresses the highest-priority tasks and computes the necessary joint torques. By using the null-space projection technique, task priorities are preserved while enabling a single optimization process via a QP solver to generate the final solution [39].

In dynamic environments, WBC adjusts torque outputs in real time to accommodate changes in both the environment and the robot’s state [30]. This process involves solving a dynamic optimization problem in each control cycle to recalculate the torques, ensuring that tasks are prioritized and managed based on updated state information. The WBC framework leverages inverse kinematics to convert the foot-end positions generated by MPC into joint angles, ensuring that task constraints are respected.

The derivation of WBC begins with optimal reaction forces for the stance legs, the base’s orientation and position, and the swing leg’s position, obtained through MPC optimization. The velocity-based WBC is then expressed using null-space projection, as shown in Equation (10):

(10) $${\dot{q}}_{i}^{\mathrm{cmd}}={\dot{q}}_{i-1}^{\mathrm{cmd}}+J_{i|\mathrm{pre}}^{†}\left({\dot{x}}_{i}^{\mathrm{des}}-J_{i}q_{i-1}^{\mathrm{cmd}}\right),\;\Delta q_{i}=\Delta q_{i-1}+J_{i|\mathrm{pre}}\left(e_{i}-J_{i}\Delta q_{i-1}\right)$$

Equation (10) shows how the pseudo-inverse of the Jacobian matrix $J_{i|\mathrm{pre}}^{†}$ calculates the required joint angles from the desired foot-end positions ${\dot{x}}_{i}^{\mathrm{des}}$ based on the IK equations. These joint angles are used to compute optimal torque commands, ensuring precise motion control while adhering to physical constraints.

In the velocity space, constraints are expressed as equality constraints, enabling their direct application. Afterwards, joint torques are calculated. To satisfy inequality constraints, such as friction cones, which are defined in the acceleration space related to the foot contact forces, null-space projection is employed. This method establishes task prioritization and generates an initial solution. A QP solver is then used to optimize acceleration and the contact forces. Relaxation variables are introduced to ensure that both dynamic and inequality constraints are met. The formulation for the acceleration-based WBC is provided in Equation (11):

(11) $${\ddot{q}}_{i}^{\mathrm{cmd}}={\ddot{q}}_{i-1}^{\mathrm{cmd}}+J_{i|\mathrm{pre}}^{\mathrm{dyn}}\left({\ddot{x}}_{i}^{\mathrm{cmd}}-{\dot{J}}_{i}\dot{q}-J_{i}{\ddot{q}}_{i-1}^{\mathrm{cmd}}\right)$$

To compute the torque commands, the QP solver minimizes the acceleration command tracking error and reaction forces, yielding optimal reaction forces while satisfying inequality constraints. The QP problem is formulated as shown in Equation (12):

(12) $$\min_{\delta_{f_{r}},\delta_{f}}\delta_{f_{r}}^{⊤}Q_{1}\delta_{f_{r}}+\delta_{f}^{⊤}Q_{2}\delta_{f}$$

subject to Equation (13):

(13) $$S_{f}\left(A\ddot{q}+b+g\right)=S_{f}J_{c}^{⊤}f_{r},\;Wf_{r}\geq 0$$

By substituting accelerations $a_{f}$ and $a_{j}$ into the multi-body kinematics model, the joint torque command $T_{j}$ is computed as shown in Equation (14):

(14) Aq¨fq¨j+b+g=06τ+Jc⊤fr

#### 3.3.3. PD Controller and Error Compensation

The initial torques generated by WBC ($\tau_{WBC}$) cannot be directly applied, as there may be deviations between the actual joint positions and velocities and the desired values during execution. To ensure that the joints accurately reach their target positions, we introduce the PD controller to perform error compensation (see Figure 5).

The PD controller generates a compensation torque based on the difference between the target joint positions and velocities and the actual values. This torque is calculated as follows:

(15) $$\tau_{PD}=K_{p}\left(q_{d}-q_{actual}\right)+K_{d}\left({\dot{q}}_{d}-{\dot{q}}_{actual}\right),$$

where −$K_{p}$ is the proportional gain, used to adjust the output torque based on position error; −$K_{d}$ is the derivative gain, used to adjust the output torque based on velocity error; −$q_{d}$ and ${\dot{q}}_{d}$ are the desired joint position and velocity, respectively; and −$q_{actual}$ and ${\dot{q}}_{actual}$ are the actual measured joint position and velocity.

**Figure 6:**
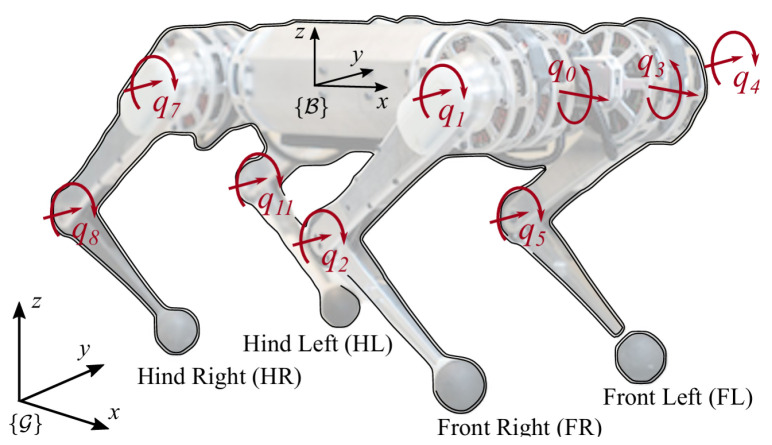
Robot coordinates and joint point settings [30].

The final joint torque command $\tau_{final}$ is a combination of the WBC-generated torque $\tau_{WBC}$ and the PD controller’s error compensation torque $\tau_{PD}$, as expressed in Equation (16), and we can see the torque distribution in Figure 6. A total of nine torque $q_{0}$ motors adjust the pitch or yaw attitude of the airframe. The remaining eight controlling motors are responsible for the support, attitude adjustment, positional movement, and swing states of the four legs.

(16) $$\tau_{final}=\tau_{WBC}+\tau_{PD}$$

This combination ensures precise joint control, where WBC handles global torque optimization, and the PD controller corrects for joint-level errors to achieve accurate motion.

## 4. Trajectory Planning

After successfully assembling the underlying motion control framework for the quadruped robot, the next focus is on the trajectory planning module. This section outlines the entire process, from environmental sensing and point cloud information acquisition to grid map generation and the complete trajectory generation and optimization workflow.

### 4.1. Sensory Information Acquisition

In this module, sensory data are primarily acquired using the Intel RealSense D435i depth camera, which is integrated with a six-DOF IMU. The combination of depth sensing and pose estimation provides the robot with high-density point cloud data.

The D435i captures depth information using infrared and RGB cameras. By projecting structured light and using triangulation, the system generates a 3D point cloud, where each point is represented by p=(x,y,z). Meanwhile, the integrated IMU provides six-DoF data: linear acceleration in three axes and angular velocity. The robot’s orientation is estimated using quaternions q=(qx,qy,qz,qw), while the IMU’s accelerometers and gyroscopes measure the robot’s linear and angular motion.

**Figure 7:**
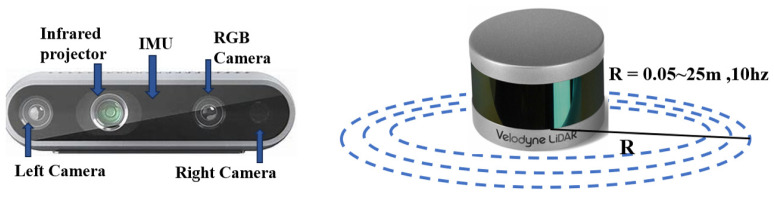
Intel D435i and velodyne LIDAR.

For comparison purposes, Figure 7 shows a velodyne LiDAR sensor, which is also incorporated into the experiments. The LiDAR is configured as a 360-degree scanning 2D sensor, with a scanning frequency of 10 Hz and a measurement range from 0.05 m to 25 m. Although the LiDAR-generated point cloud is sparser than that of the D435i, it performs well in detecting distant obstacles [40].

### 4.2. Grid Map Construction

After obtaining the point cloud data and pose information from the camera, the next step involves constructing a real-time updated grid map. This section explains the 3D-3D pose estimation process and the grid map generation method, which forms the foundation for robot navigation and mapping.

#### The 3D-3D Pose Estimation

**Figure 8:**
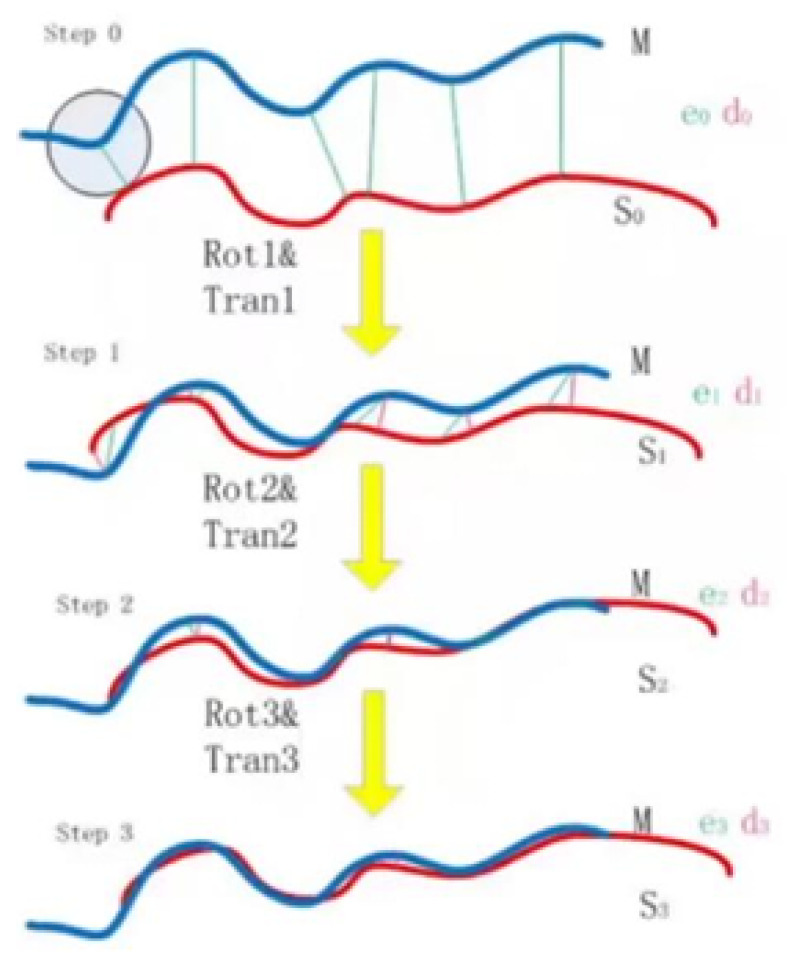
ICP diagram.

The Iterative Closest Point (ICP) algorithm is employed to match the point clouds between different frames. This algorithm iteratively refines the alignment of two point clouds by minimizing the distance between corresponding points. The ICP process begins with an initial guess of the rotation (*R*) and translation (*T*), as demonstrated in the pseudocode shown in Algorithm 1 and the corresponding diagram in Figure 8. In each iteration, a point from the first point cloud is matched with the nearest point in the second point cloud, and the transformation is updated based on these correspondences. The iteration continues until the error converges to a minimal value or the maximum number of iterations is reached, resulting in an aligned point cloud.
**Algorithm 1** ICP Processing Flow1: **Initialize:** Initial Guess for Rotation *R* and Translation *T*2: **while** error not converged or max iterations not reached **do**3: For each point $p_{1}$ in PointCloud1, find the nearest point $p_{2}$ in PointCloud24: Compute the difference between corresponding points5: Update rotation *R* and translation *T* based on the computed differences6: **end While**7: Output the final aligned PointCloud

During data association, the nearest neighbor search is typically used to find the corresponding points between two point clouds. This approach calculates the Euclidean distance for each point, which has a computational complexity of O(N). However, when the same dataset is queried repeatedly, the Kd-tree data structure can be employed to optimize the search, reducing the complexity to O(logN). This optimization reduces the computational load, making the process more efficient for large datasets.

### 4.3. Grid Map Construction and Inflation Handling

Before constructing the grid map, point cloud data are captured using a depth camera, and the map is stored in an OccupancyMap format, where each cell is marked as either free (0) or occupied (1). To simplify computations, it is assumed that the map is static and that each cell is independent. While this method places significant memory pressure on the processor and cannot store continuous obstacle information efficiently, it remains effective for planar obstacle detection in ground-based robot experiments.

**Figure 9:**
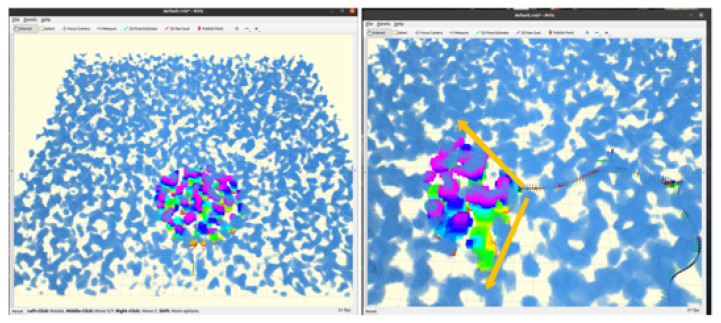
Comparison of before and after modifying the perception region.

After generating the grid map, local inflation is applied to ensure safe obstacle avoidance. We modify the parameter ‘obstacleinflation=0.5’, and the map inflates around obstacles, ensuring that the robot maintains a safe distance during path planning. Figure 9 shows the initial grid map, and Figure 9 shows the map after inflation. The inflation algorithm focuses on the robot’s forward field of view, which is approximately 60 degrees, optimizing processing by ignoring unnecessary regions. After inflation, the perception module ensures a sufficient safety margin to avoid collisions during navigation.

### 4.4. Sensory Information Communication in ROS

During the grid map generation process, techniques such as point cloud preprocessing and overlap detection were utilized to manage the high-density data generated by the depth camera. These point cloud data, significantly more complex than the LiDAR data, required downsampling to alleviate system processing pressures. The unordered multidimensional array format of the point cloud posed computational challenges during map rendering.

To improve efficiency, a KD-tree was used to associate the data, simplifying the subsequent processing steps. The primary implementation of this process is handled by the pcl_cloud_render.cpp file, which processes raw point cloud data from the grid map. PCL (Point Cloud Library) rendering applies downsampling and density reduction, ensuring that the processed point cloud data conform to the required format for robot perception.

We use a KD tree to build the data structure, facilitating fast nearest neighbor searches. And the PCL was used for downsampling and density reduction, ensuring that the processed point cloud data conform to the required format for the perception system.

**Figure 10:**
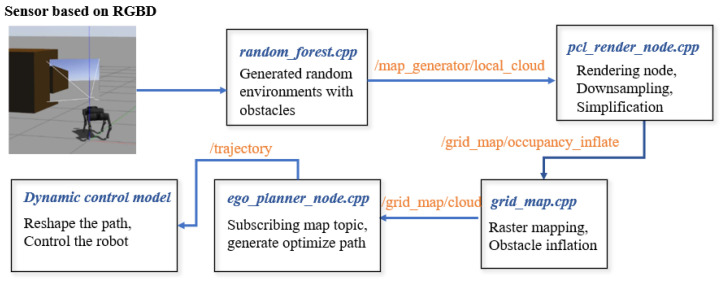
Point cloud processing flowchart.

After preprocessing, the filtered point cloud is returned to the grid map for further inflation. The inflated map is then published to the ROS topic grid_map/occupancy_inflate, allowing the quadruped robot’s 2D EGO-Planner to use the map for trajectory planning. As shown in Figure 10, the system subscribes to relevant topics, processes sensory data, and performs obstacle inflation. The expanded grid map, along with the original PCL data, is broadcast in PCL format. With the completion of the grid map generation and obstacle inflation processes, the system is now fully prepared for trajectory search and execution.

### 4.5. EGO-Planner

In this section, we provide a detailed overview of the complete trajectory generation process. We incorporate modifications to the 2D EGO-Planner to meet the robot’s requirements for planar motion. The overall planning framework employs a multi-layered path planning approach, which includes both global path planning and local trajectory optimization.

#### Trajectory Generation Process

In the global path planning phase, the system employs a dynamic A* algorithm to generate an initial global path. This algorithm operates on a grid map to produce a rough path from the start point to the target destination. Although this path does not fully account for the robot’s dynamic constraints, it effectively avoids the static obstacles present in the initial grid map, thus providing directional guidance for subsequent local optimization. The resulting discrete path points serve as the initial input for the EGO-Planner, which will perform further local refinements.

In the local path planning phase, the EGO-Planner refines the initial path using real-time environmental data and the trajectory provided by the A* algorithm [41]. The primary function of the EGO-Planner is to generate a B-spline curve that smooths and optimizes the path while ensuring collision avoidance [42]. When a control point detects a collision with an obstacle, the system employs gradient descent to move the point away from the obstacle.

**Figure 11:**
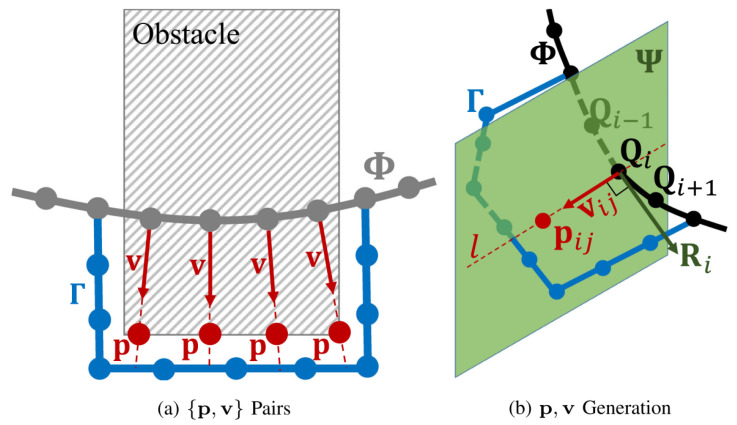
{p, v} generation: (**a**) the creation of {p, v} pairs for collision points; (**b**) the process of generating anchor points and repulsive vectors for dynamic obstacle avoidance [41].

Specifically, as illustrated in Figure 11a, for each collision point, an anchor point $p_{ij}$ is generated on the obstacle surface. The system then calculates a repulsive vector $v_{ij}$ that directs the control point away from the obstacle, pushing it to a safer location: $v_{ij}=f\left(p_{ij},Q_{i}\right),$ where $p_{ij}$ is the anchor point on the obstacle surface, and $Q_{i}$ is the collision-detected control point. This dynamic update of the {p,v} pairs allows the trajectory to be iteratively adjusted, forming a collision-free path.

Figure 11b further illustrates the process of generating anchor points $p_{ij}$ and repulsive vectors $v_{ij}$ in a 3D space. The control point $Q_{i}$ is first identified as a collision point. In a 3D space, the anchor point $p_{ij}$ is positioned on the surface of the obstacle, close to the projection of the control point $Q_{i}$ along the path Γ. The system then calculates the repulsive vector $v_{ij}$ from the control point to the anchor point, thereby modifying the position of $Q_{i}$ to ensure that it moves away from the obstacle.

**Figure 12:**
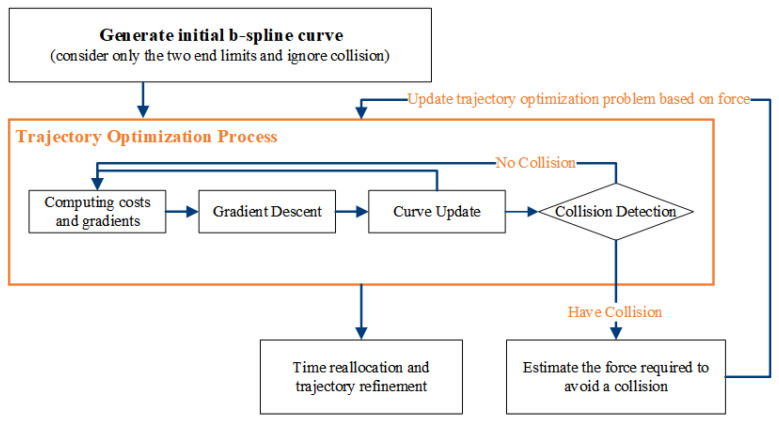
Overall framework of 2D EGO-Planner.

To handle complex terrains and dynamic obstacles, the EGO-Planner continuously updates the trajectory’s control points based on real-time sensory input. As shown in Figure 12, this adjustment process is not a one-time event but involves multiple iterations based on continuous feedback from the environment. During each iteration, the system performs collision detection, cost computation, and curve updates until the path satisfies the collision avoidance requirements.

To meet both spatial and dynamic requirements, the EGO-Planner incorporates a time reallocation mechanism. As shown in Figure 12, this mechanism optimizes the time intervals Δt between control points $\left\{Q_{1},Q_{2},\ldots ,Q_{N_{c}}\right\}$ based on the computed derivatives (velocity $\dot{Q}\left(t\right)$, acceleration $\ddot{Q}\left(t\right)$, and jerk $\dddot{Q}\left(t\right)$) at each time step. By adjusting these intervals, the trajectory maintains smoothness while adhering to dynamic feasibility constraints. The control points’ velocities ${\dot{Q}}_{i}$ correspond to each time interval $\Delta t_{i}$, ensuring that changes in speed, acceleration, and jerk are within the system’s limits.

Once the control points have been adjusted to avoid obstacles, the system proceeds to merge these points with the unaffected segments of the original path. This merging process takes advantage of the convex hull property of B-splines to ensure a smooth transition between the modified and original segments.

In the EGO-Planner, the trajectory is modeled using uniform B-spline curves ϕ. These curves are defined by the order $p_{b}$, the control points $\left\{Q_{1},Q_{2},\ldots ,Q_{N_{c}}\right\}$, and a knot vector $\left\{t_{1},t_{2},\ldots ,t_{M}\right\}$, where $Q_{i}\in R^{3}$, $t_{m}\in R$, and $M=N_{c}+p_{b}$. Each node of the B-spline curve has an equal time interval, $\Delta t=t_{m+1}-t_{m}$. The convex hull property of B-spline curves allows for a smooth merging of the modified and original path segments during trajectory optimization.

The dynamic properties of the trajectory, including velocity $\dot{p}$ and acceleration $\ddot{p}$, are derived directly from the control points of the B-spline curve. To ensure that the trajectory satisfies dynamic constraints, the EGO-Planner uses a time reallocation mechanism, adjusting the time intervals Δt between the control points to regulate velocity $\dot{p}$, acceleration $\ddot{p}$, and jerk, maintaining smoothness and dynamic feasibility.

### 4.6. Improvements in EGO-Planner

#### The 2D EGO-Planner

In practice, the A* algorithm is confined to searching within a 2D plane, simplifying the path generation process. However, the obstacle detection module operates with a 3D grid map, which accurately represents the surrounding environment. The workflow of the 2D EGO-Planner begins by initializing control points $Q=\{Q_{1},Q_{2},\ldots ,Q_{n}\}$ in a 2D space to form the initial path. While the planner generates the path in 2D, the obstacle detection module continuously updates the 3D grid map using real-time sensor data to identify obstacle segments S. Algorithm 2 shows that, for each detected obstacle segment, the system computes repulsive forces, generating anchor points $p_{ij}$ and repulsive vectors $v_{ij}$. These forces are then used to optimize the B-spline curve in the 2D plane. In this 2D context, the system evaluates dynamic constraints by computing the first and second derivatives of the B-spline control points:

(17) $$V_{i}=\frac{Q_{i+1}-Q_{i}}{\Delta t},\;A_{i}=\frac{V_{i+1}-V_{i}}{\Delta t}.$$

The optimization process iteratively adjusts both the control points and the time intervals Δt to ensure compliance with velocity and acceleration constraints.
**Algorithm 2** 2D-EGO-Planner for Ground Robots1:Initialize: **Q** (Control Points in 2D), **Env** (Environment), **goal**2:**while** not reached goal **do**3:    **S** ← Detect Obstacle Segments in 2D4:    **for all** segments in **S do**5:        **p, v** ← Find Repulsive Force (**S**)6:        Update Control Points: **Q** ← Optimize B-spline trajectory in 2D7:    **end for**8:    **if** Trajectory violates dynamic constraints **then**9:        Adjust Time Allocation and Refine Trajectory10:    **end if**11:    Move to next waypoint12:**end while**

During each iteration, a feedback loop continuously evaluates the trajectory for violations of dynamic constraints. The control points are refined to ensure dynamic feasibility, while the time intervals are reallocated when necessary, maintaining an optimized path until the target point is reached.

## 5. Results

### 5.1. Robot Assembly

**Figure 13:**
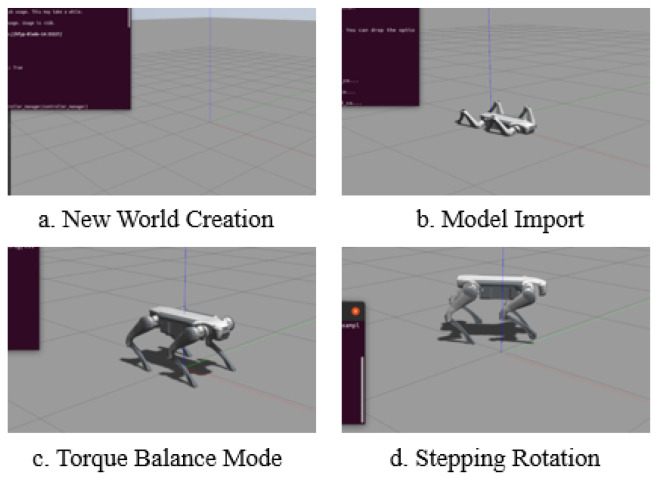
Robot initialization and control process in Gazebo simulation: (**a**) Gazebo environment creation, (**b**) robot model import, (**c**) torque balance mode activation, and (**d**) robot stepping and rotation in simulation.

After the establishment of the ROS node communication system, we can build the robot dog model while completing some simple movement practices. The robot initialization process is shown in detail in Figure 13. Firstly, the world model is launched to create a Gazebo simulation environment; then, we input the robot model into the simulation. Afterwards, the torque balance command is given by a keyboard, and the commands are transmitted to the actuator via the serial communication protocol. The actuator adjusts the joint torque according to the received commands to ensure that the robot dog can remain stable on different surfaces. Lastly, the trot command is given by a keyboard command. At this time, the robot dog will follow the speed given in the command velocity topic to control the CoM to reach the target position and orientation. During operation, by reading the information on the terminal, it can be seen that the robot dog’s delay is less than 25 ms.

### 5.2. Robot Control

**Figure 14:**
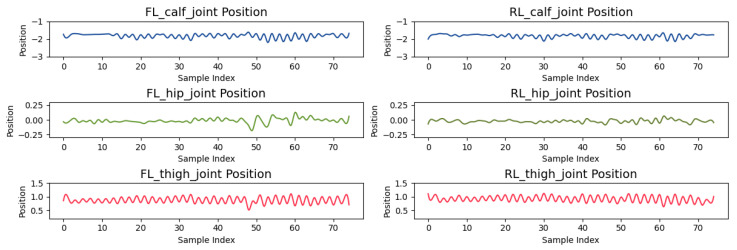
Joint rotational angles of FL and RL legs.

The experiment in this section analyzes the motion control of the quadruped robot’s front-left (FL) and rear-left (RL) leg joints, focusing on the distribution of the CoM trajectory. The joint positions, as shown in Figure 14, exhibit periodic oscillations, demonstrating the regularity of leg movements during the gait cycle. For example, the FL_thigh_joint oscillates between 0.5 and 1.5 radians, while the FL_calf_joint shows a larger range from −2.5 to −1.0 radians, indicating more significant motion during transitions. This periodic behavior is similarly observed in other joints, clearly reflecting the different stages of the gait cycle.

The following figures present data from an experiment in which the quadruped robot autonomously navigated a simple environment, successfully avoiding static obstacles. During this experiment, the robot performed a basic turning maneuver while maintaining stability and control, as reflected in the joint positions, velocities, and torques.

Figure 14 shows that the joint positions of the front-left (FL) and rear-left (RL) legs exhibit periodic oscillations, typical of a gait cycle. For example, the FL_thigh_joint oscillates between 0.5 and 1.5 radians, while the FL_calf_joint shows a larger range from −2.5 to −1.0 radians, indicating more significant motion during stance and swing transitions. This behavior is consistent across other joints, reflecting the different phases of the gait cycle.

**Figure 15:**
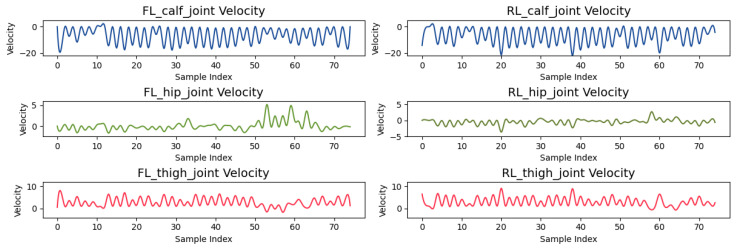
Joint angular velocities of FL and RL legs.

Joint angular velocities, while still periodic, exhibit smaller fluctuations (Figure 15). The RL_calf_joint oscillates between −20 and 0 rad/s, peaking during leg lifting and lowering, indicating intensive movement. The FL_thigh_joint, however, remains more stable, oscillating between 0 and 10 rad/s, reflecting its role in providing steady support.

**Figure 16:**
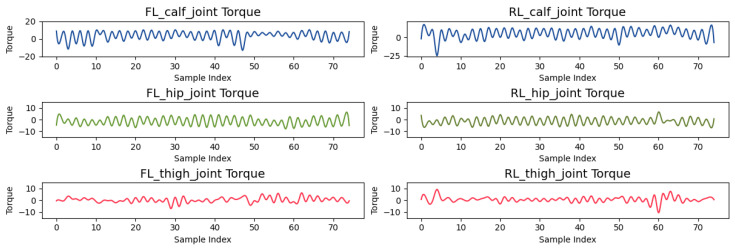
Torque applied to FL and RL joints during the gait cycle.

Torque, as shown in Figure 16, provides further insight into external loads during motion. The FL_calf_joint experiences torque variations from −25 to 20 Nm, reflecting the load changes during leg lifting and weight bearing. In contrast, the RL_hip_joint shows relatively smaller torque variations, maintaining stability throughout the gait.

These results indicate that the robot’s motion control system efficiently allocates the CoM trajectory to the joints, allowing for stable and controlled gait execution across various phases of the cycle.

### 5.3. RGB-D-Based Autonomous Navigation in Static Environment

**Figure 17:**
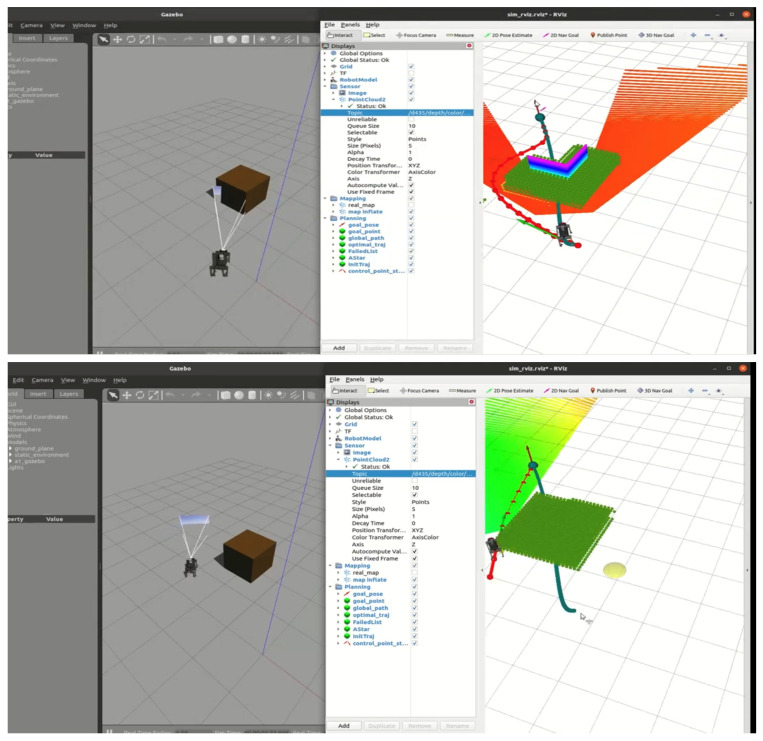
The robot navigating in a simple environment using a camera.

**Figure 18:**
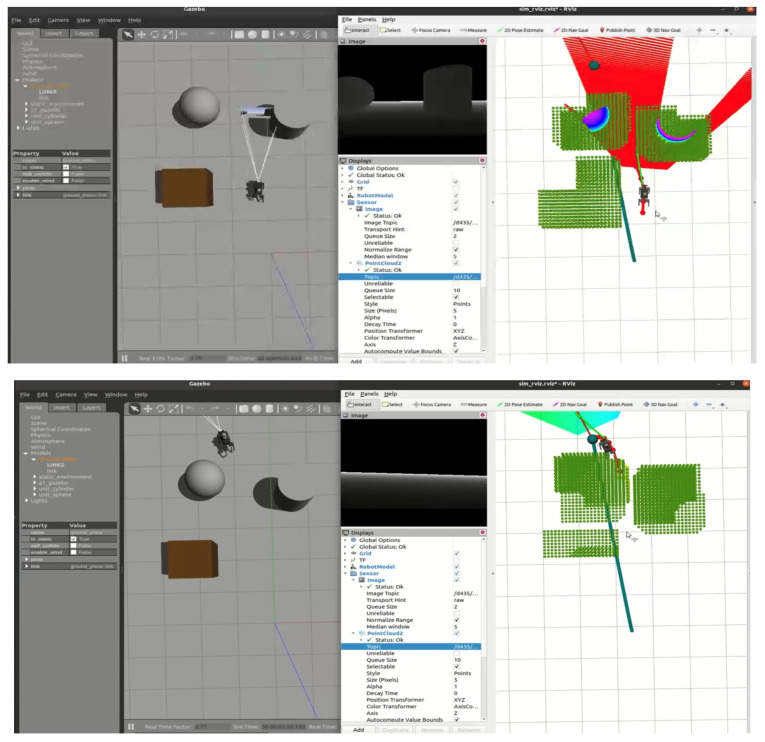
The robot navigating in a complex environment using a camera.

This section introduces the RGB-D-based autonomous navigation. Figure 17 and Figure 18 illustrate the initial path planning and dynamic replanning process when encountering simple obstacles. In the initial state, the path planner generates a baseline trajectory without considering collisions. As the robot moves along this trajectory and detects obstacles, the state machine transitions from the EXEC_TRAJ state to the REPLAN_TRAJ state, prompting the planner to update the CoM trajectory to ensure obstacle avoidance.

Based on the preliminary D* path, the 2D EGO-Planner computes a safe trajectory around the detected obstacles. Figure 17 shows the robot’s progress through the environment, successfully navigating towards the target position while avoiding obstacles. As shown in Figure 18, the Gazebo interface shows how the D435i camera mounted on the robot detects the obstacles in real time. The system performs real-time replanning when necessary, with an average of 35 iterations per robo_plan, typically completing the entire navigation process, including 200 iterations of robo_plan, within 15 s. Due to the limitations of the experimental platform, prolonged operation may lead to system instability. Therefore, we constrained the robot’s movement to a small but characteristic environment with distinct obstacles to evaluate its performance. Despite the limited time frame, the experiment demonstrates a complete autonomous navigation process, showcasing the robot’s ability to efficiently avoid obstacles and adjust its trajectory in real time.

Additionally, we optimized the robot’s velocity limits to ensure stability during execution. The velocity thresholds were set at $v_{x},v_{y}=0.5\;m/s$, as higher thresholds led to instability. This configuration provided an optimal balance between speed and navigation safety.

**Figure 19:**
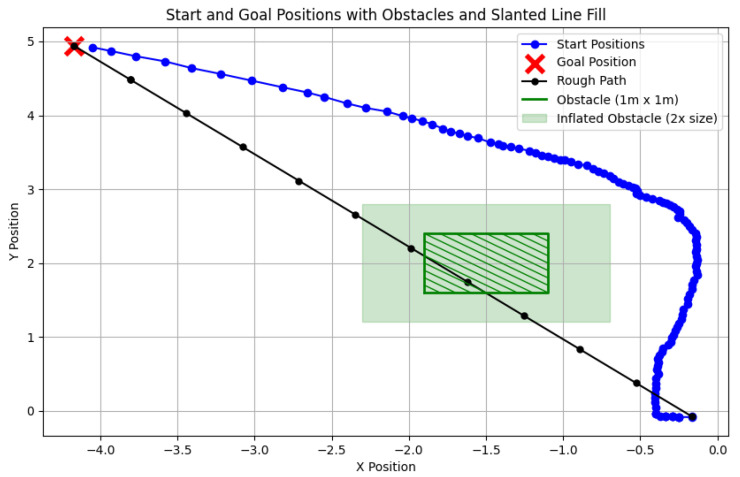
A 2D trajectory showing start and goal positions, obstacles, and rough path.

Figure 19 illustrates the complete trajectory in a 2D plane. Initially, a rough path (black line) is generated based on the start and goal positions. The green shaded rectangle indicates an artificially set obstacle and its inflated boundary, which aligns with the obstacles in the Gazebo simulation. The red points represent predefined target points, while the blue points denote the planner’s output path.

The robot dynamically adjusts its path to avoid collisions, maintaining the replanning time for each iteration within 10 milliseconds to ensure real-time performance. Each iteration represents the time it takes for the robot, in the robo_plan state, to compute the trajectory points for the next time step. Given that a typical experiment involves multiple instances of this replanning process, this demonstrates the system’s ability to consistently perform real-time trajectory adjustments. The red points represent predefined target points, while the blue points denote the planner’s output path.

Notably, the trajectory points displayed include all coordinate points from each replanning step rather than those being sampled. This approach highlights the frequency of planning updates, particularly during the initial phase when the robot requires more time to determine a suitable direction near its starting position. As the trajectory stabilizes and no further obstacles are detected, the robot moves smoothly, reducing the need for frequent replanning and lowering the computational load.

The experiment also demonstrates that the size of the obstacle’s inflated boundary significantly influences the replanning frequency. A larger inflation area results in more frequent replanning, reflecting the planner’s sensitivity to dynamic obstacles and its path adjustment capabilities.

### 5.4. RGB-D-Based Autonomous Navigation in Dynamic Environment

**Figure 20:**
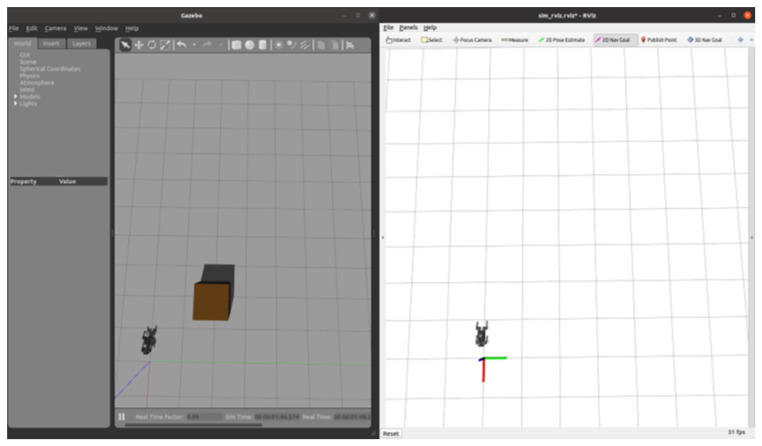
Initial environment setup.

**Figure 21:**
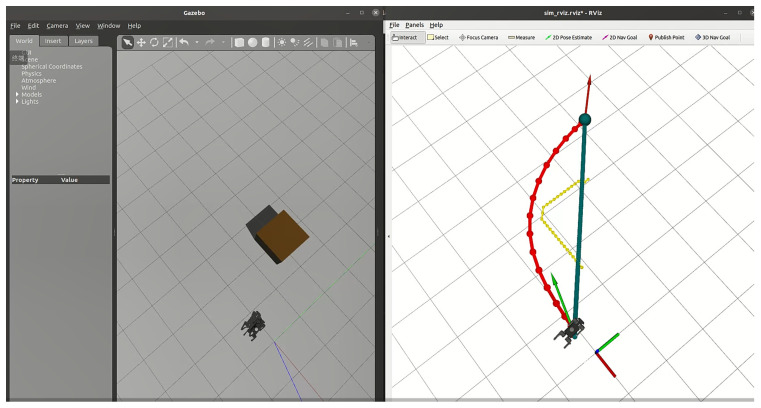
The robot starts navigating in a simple environment with a static obstacle (brown box).

In this section, we introduce dynamic obstacles into the simulation environment. As shown in Figure 20, the brown box in the initial environment represents a static obstacle, and the robot dog was originally planned to bypass it along the designated path towards the target point. To validate the system’s robustness and the local planner’s real-time planning capability, we manually added a sudden dynamic obstacle in the Gazebo environment, as shown in Figure 21. Upon detecting the obstacle, the planner immediately recalculates the path, generating a new trajectory to bypass the obstacle and continue towards the target point.

**Figure 22:**
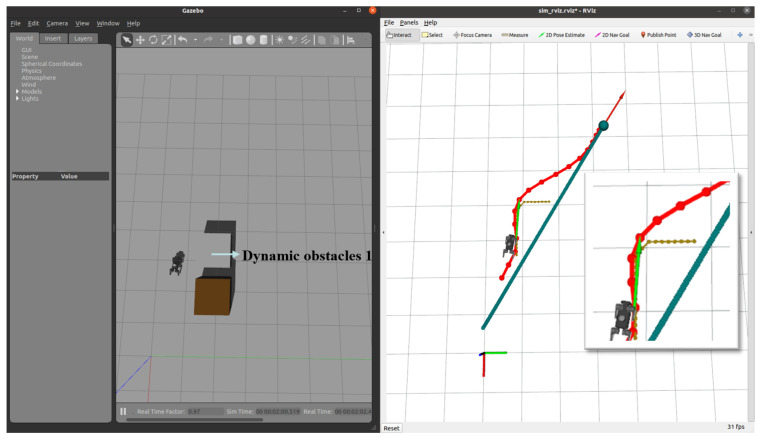
Dynamic Obstacle 1 introduced: the robot detects a new obstacle and recalculates its path.

**Figure 23:**
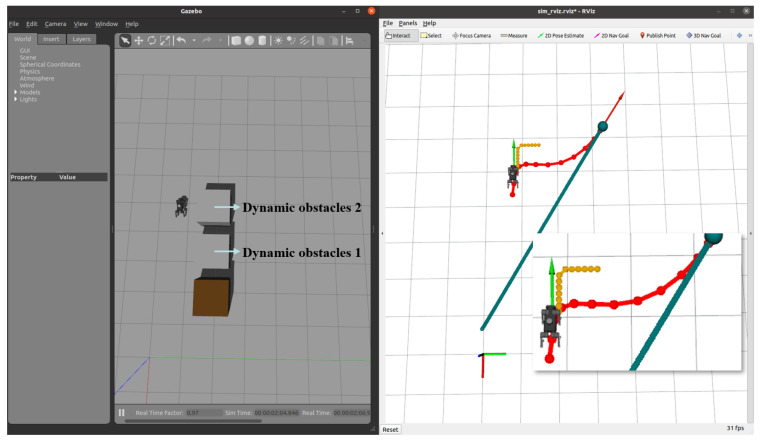
Dynamic Obstacle 2 introduced: after avoiding the first obstacle, a second obstacle is introduced and detected by the planner.

**Figure 24:**
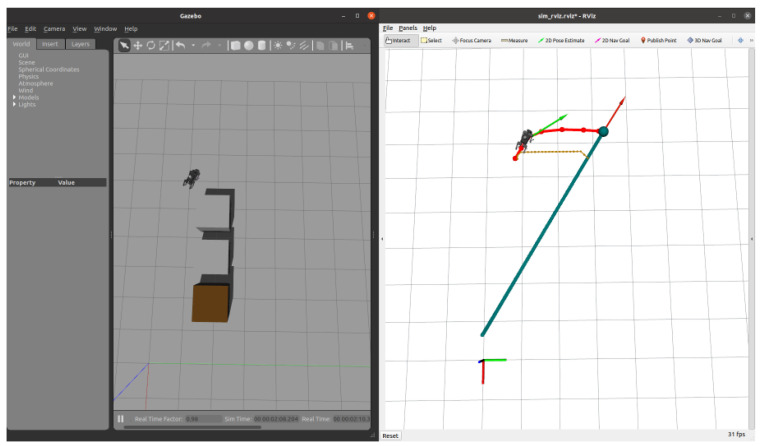
Approaching the target: the robot adjusts its path to approach the target point as the distance shortens.

**Figure 25:**
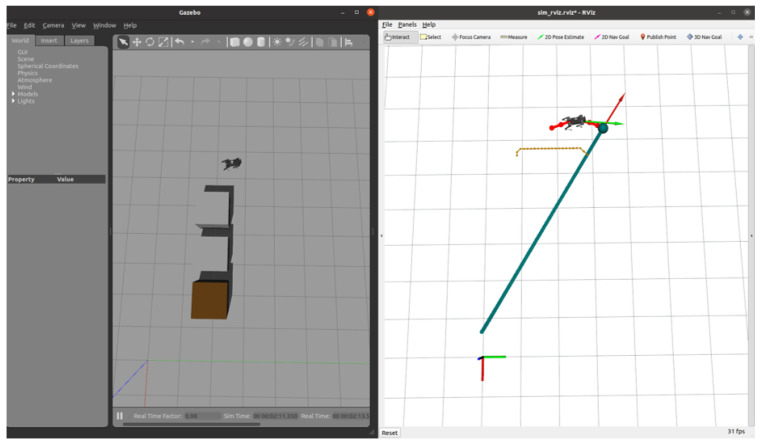
Reaching the target: the robot completes its path and reaches the designated target point.

Subsequently, after the robot dog stabilized along the new path, we introduced a second dynamic obstacle along its trajectory, as shown in Figure 22. The EGO-Planner detected the new obstacle and updated the trajectory to avoid it. As shown in Figure 23, Figure 24 and Figure 25, as the robot gradually approached the target point, the distance covered by each planning iteration shortened, and the trajectory steadily converged towards the goal. Ultimately, the robot dog successfully avoided all obstacles and reached the target point without issue.

**Figure 26:**
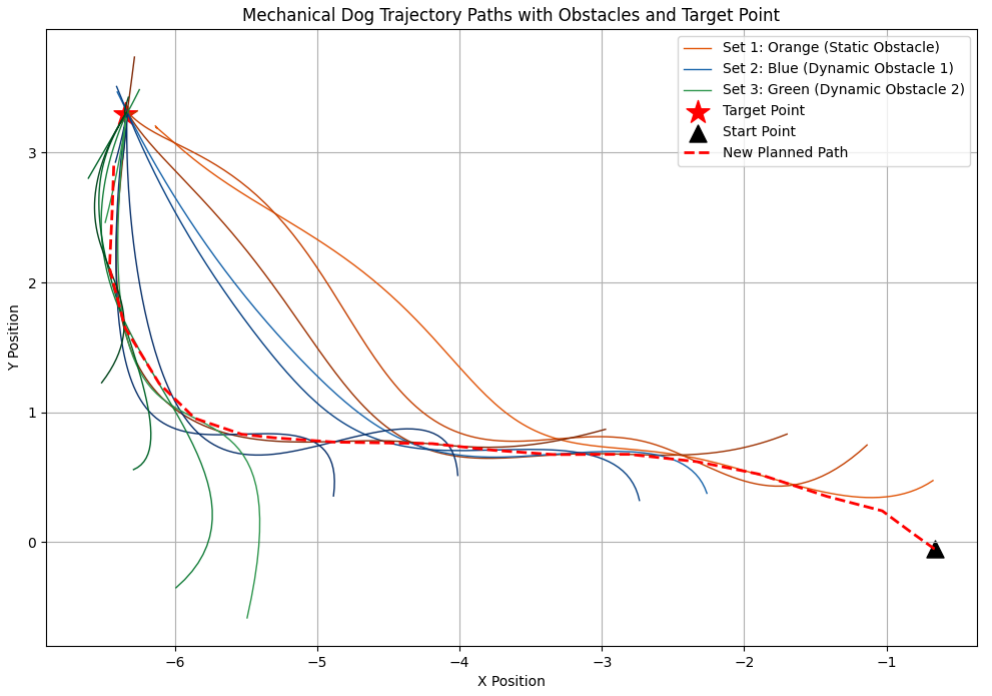
Real-time B-spline trajectory updates in response to dynamic obstacles. Set 1 (orange) shows the initial path avoiding static obstacles. When the first dynamic obstacle is detected, the EGO-Planner updates the path (Set 2, blue) using local optimization. A second obstacle prompts another adjustment (Set 3, green), guiding the robot smoothly towards the target as trajectory updates become more frequent.

Additionally, we demonstrate the real-time updates of the planner’s B-spline curve during the robot’s motion in response to dynamic obstacles. As Figure 26 shows, Set 1 (orange curves) depicts the initial trajectory generated by the planner as the robot avoids static obstacles. Each trajectory update corresponds to a new B-spline curve generated as the robot advances. The gradient from light to dark colors reflects the increasing index of trajectory updates over time.

When the first dynamic obstacle appears along the planned path, the EGO-Planner immediately updates the trajectory, as shown by Set 2 (blue curve) in Figure 26. The depth camera detects the newly emerged obstacle and updates the grid map accordingly. Using gradient projection, the 2D-EGO-Planner applies gradient-based local optimization to pull the collided segments onto the surface of the obstacle, adjust the original path, and update the B-spline curve to avoid the new obstacle.

Subsequently, the robot dog quickly stabilizes its motion along the newly planned trajectory. After a 2 s interval, we manually introduce a second dynamic obstacle along the newly planned trajectory (blue curve). Upon detecting the new obstacle, the EGO-Planner quickly updates the trajectory to avoid it, resulting in a sudden shift in the B-spline curve. Set 3 (green curves) illustrates this adaptive behavior.

As the robot advances towards the target point, the distance covered by each planning iteration gradually shortens, and the trajectory steadily converges towards the goal. The planner’s iterative adjustments ensure smooth and efficient navigation, ultimately guiding the robot to reach the target point without collisions.

### 5.5. SLAM-Based Autonomous Navigation

In this section, we implement path planning using SLAM and describe the integration of LIDAR data with inertial sensors for precise navigation. Velodyne LIDAR is used to capture 360-degree point cloud data, providing a comprehensive view of the environment. SLAM offers a distinct advantage over the RGB-D system by generating a denser, 360-degree environmental map. However, this increased data density also introduces challenges in terms of data processing and filtering.

In the experiment, we optimized the data processing pipeline by modifying the ROS topic to filter out irrelevant points before transmitting them to the pcl_renderer. This approach focused on transmitting only critical point cloud data, reducing computational overhead. Despite these optimizations, SLAM still incurs higher processing costs than RGB-D due to the volume of data. Many of the points captured, such as those behind the robot or outside the trajectory path, are not relevant to immediate navigation but still require processing.

**Figure 27:**
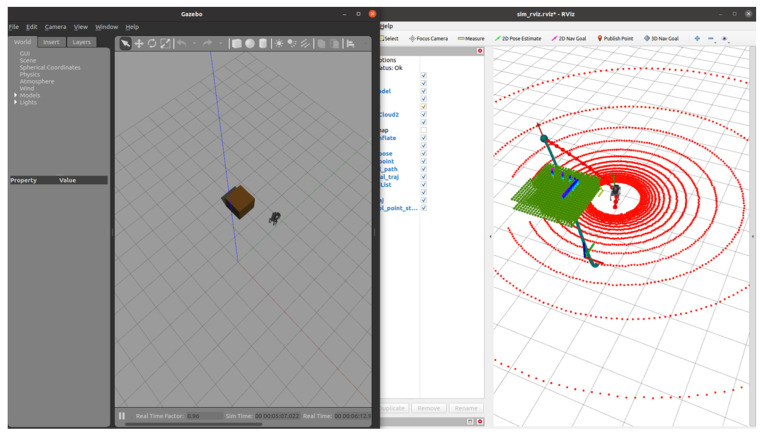
The robot navigating a simple environment using SLAM.

**Figure 28:**
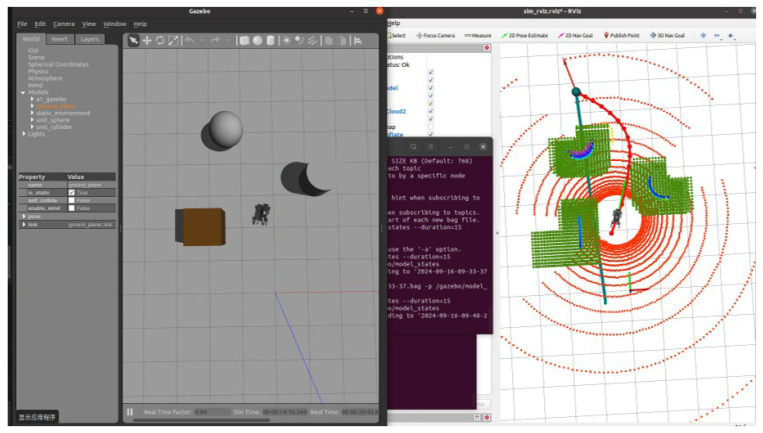
The robot navigating a complex environment using SLAM.

Figure 27 and Figure 28 show the robot navigating simple and complex environments. The system continuously updates the map and adjusts the robot’s trajectory in response to dynamic obstacles. By leveraging the expanded point cloud data, the system refines the navigation path without significant delays.

**Figure 29:**
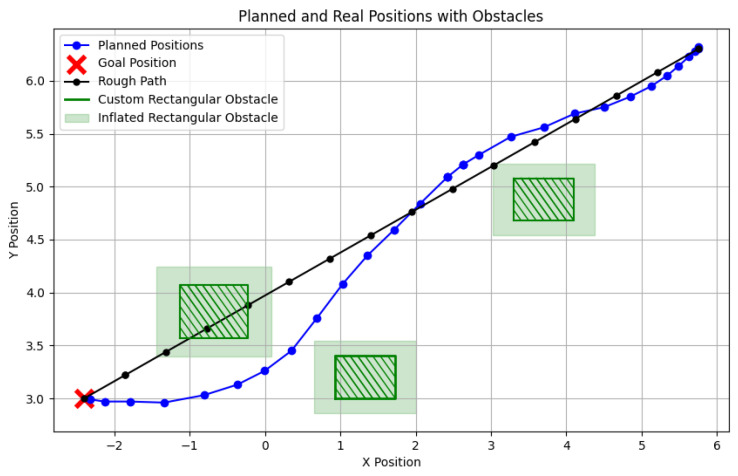
A 2D trajectory showing start and goal positions, obstacles, and the planned path in a complex environment using SLAM.

Figure 29 presents the planned trajectory and real-time adjustments made by the SLAM system. The robot starts with a rough initial path (black line), which is refined based on updated point cloud data as the robot progresses. The green rectangles represent artificially placed obstacles and their inflated boundaries. Notably, the data points displayed in the figure are sampled, with every 20 rebo_plan steps taken into account. This ensures clarity in the trajectory visualization by filtering out overly dense data from the initial planning phases. The system performs continuous replanning to ensure obstacle avoidance and path optimization.

In summary, SLAM offers more accurate path planning but operates more slowly due to the computational burden of processing high-density point cloud data. Unlike RGB-D, LIDAR captures a broader range, including irrelevant data from behind the robot and outside its trajectory. The need to filter out these extraneous points significantly increases both planning and computation times.

### 5.6. Trajectory Deviation Calculation and Comparison

**Figure 30:**
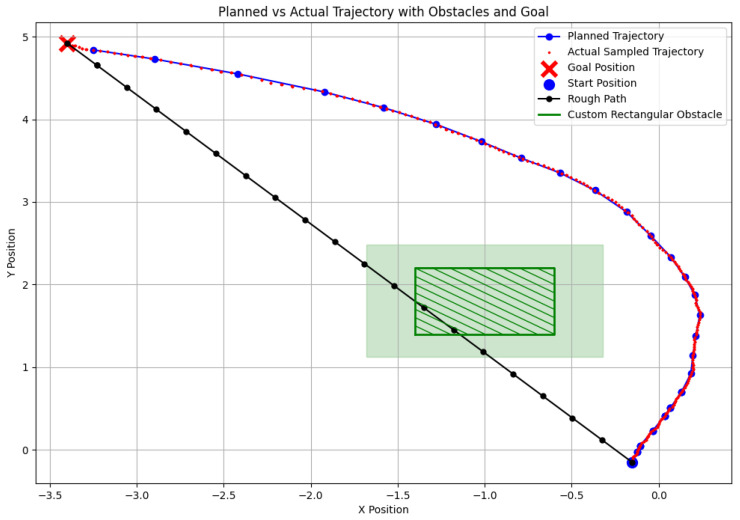
Navigation based on RGB-D camera.

**Figure 31:**
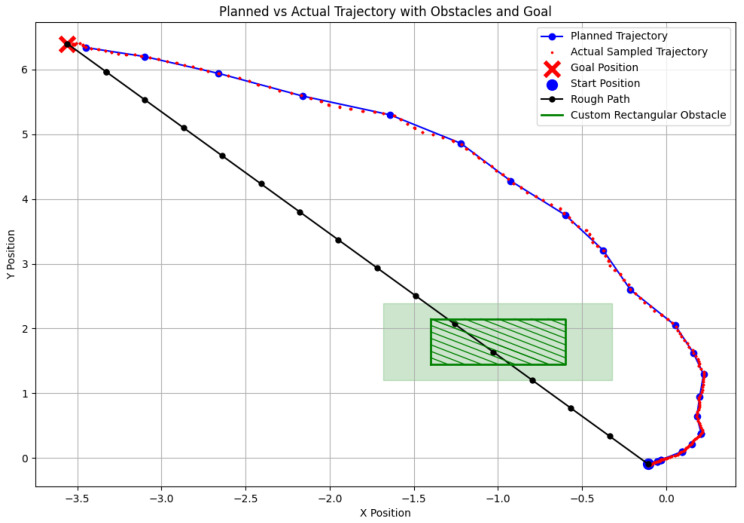
Navigation based on SLAM.

In this section, we compare the deviation between the planned trajectory and velocity from the EGO-Planner and the actual trajectory executed by the robot (see Figure 30 and Figure 31). In the experiments, the actual trajectory was sampled at ten times the frequency of the planned trajectory, resulting in multiple actual points around each planned point. By analyzing the figures, we highlight the differences in path tracking accuracy between the RGB-D and SLAM navigation systems, and we visualize the execution of both trajectories in Figure 17 and Figure 27, respectively.

Figure 30 illustrates the trajectory during RGB-D-based navigation. The robot’s trajectory is smooth and closely aligns with the planned path. Especially in the initial phase, the high-resolution environmental perception provided by the depth camera results in densely distributed trajectory points. This allows the robot to detect and avoid obstacles swiftly and effectively.

In contrast, Figure 31 shows the trajectory of the SLAM-based system. There is greater deviation early on, where the robot initially attempts a left turn before quickly correcting to the right. This behavior is caused by the sparse point cloud data from the SLAM system. In the early phase of movement, the lack of detailed environmental data leads to uncertainty and larger deviations. As more information is gathered, the robot gradually corrects its path, but the initial deviation remains significant.

### 5.7. Velocity Deviation Calculation and Comparison

**Figure 32:**
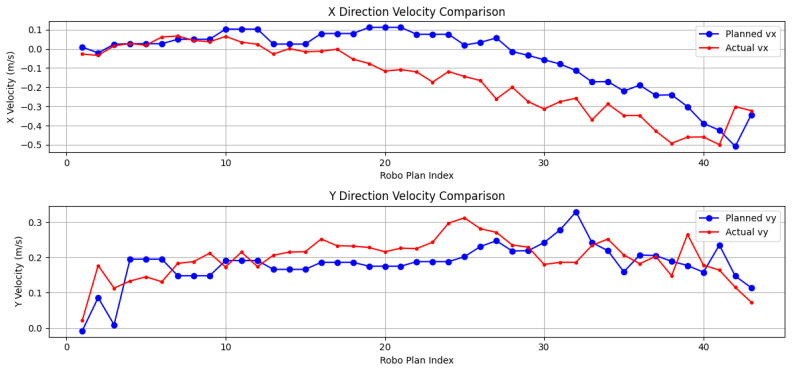
Velocity deviation based on RGB-D camera.

**Figure 33:**
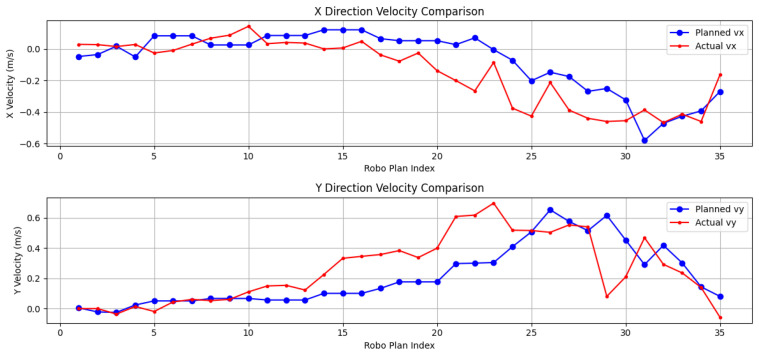
Velocity deviation based on SLAM.

This section analyzes the velocity deviations observed during the robot’s motion, comparing the performance of the RGB-D and SLAM navigation systems (Figure 32 and Figure 33). In the figures, it is clear that the velocity transitions in the RGB-D system are notably smoother, particularly in the early phases of motion. This is attributed to the high-resolution depth data provided by the RGB-D camera, allowing the robot to maintain stable forward velocity. Both $v_{x}$ and $v_{y}$ show relatively consistent trends, and the robot quickly compensates for obstacles with minimal velocity adjustments. Notably, between planning steps 15 and 25, a gradual decrease in actual $v_{x}$ can be observed, likely indicating the robot intentionally decelerating in response to a more complex terrain. At planning step 20, the actual $v_{y}$ shows a spike, representing velocity compensation during directional adjustment.

In contrast, the SLAM system exhibits larger velocity deviations, particularly in the first 10 planning steps, where $v_{x}$ decreases significantly, even turning negative. This suggests that the robot struggled with sparse point cloud data in the initial path planning phase, resulting in delayed responses to environmental changes. After the 15th planning step, $v_{y}$ actual values surpass the planned values, indicating significant velocity compensation as the robot adjusts its trajectory in response to sparse SLAM data.

### 5.8. Trajectory and Velocity Analysis

The comparative data in Table 2 highlight the performance differences between the RGB-D and SLAM systems. The planned path length of the RGB-D system ($L_{planned}$: 8.1084) is longer than that of SLAM ($L_{planned}$: 6.8821), reflecting its broader field of view and higher-resolution environment perception. However, the RGB-D system maintains a smaller path length deviation ($D_{path}$: 0.2797 vs. 0.1777), indicating more accurate path tracking.

In terms of velocity, the RGB-D system has a lower standard deviation of velocity deviations ($\sigma_{v}$: 0.0750) than SLAM ($\sigma_{v}$: 0.0771), reflecting more stable velocity control. Moreover, the cumulative velocity error is significantly lower for RGB-D ($E_{v}$: 3.8958) than for SLAM ($E_{v}$: 6.1356), further demonstrating the advantages of RGB-D in providing consistent and precise velocity adjustments.

The data show that, while both systems provide reasonable path following, the RGB-D system demonstrates better overall control, particularly in terms of velocity consistency and cumulative error. SLAM, though capable of handling dynamic environments, shows higher velocity deviations and a greater difficulty in maintaining consistent speed, especially during the early motion phases due to sparse environmental data. In summary, RGB-D outperforms SLAM in both trajectory precision and velocity stability, offering smoother navigation and reduced compensation needs during complex maneuvers.

### 5.9. Cumulative Iterations and Success Rate Analysis

**Figure 34:**
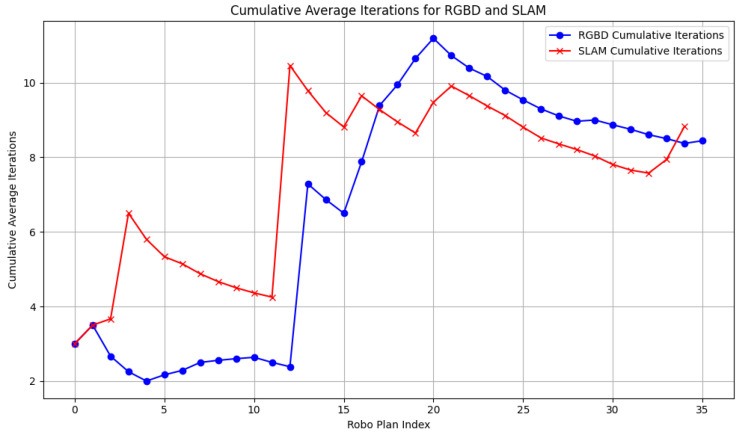
Cumulative average iterations.

The cumulative average iterations (Figure 34) reveal that the RGB-D system requires fewer iterations overall, ranging between 2 and 4, with a brief peak of 10 at step 15. This indicates its generally efficient path planning, with only minor fluctuations. In contrast, SLAM’s cumulative iterations exceed 10 during the initial stages, reflecting difficulties due to sparse point cloud data. Although SLAM stabilizes after step 20, its overall iteration count remains higher than that of RGB-D, highlighting its greater computational demand.

**Figure 35:**
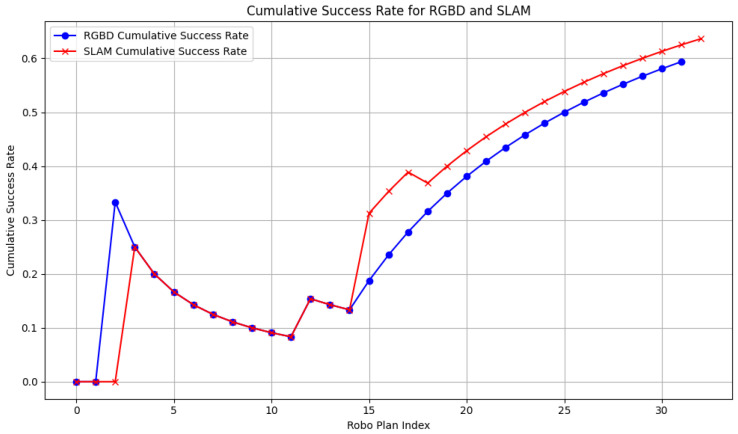
Cumulative success rate.

In terms of the cumulative success rate (Figure 35), RGB-D quickly reaches a 30% success rate by step 5, although it dips slightly before recovering to 60% by the end of the experiment. SLAM, however, struggles early on, achieving minimal success in the first 10 steps. However, as the environment becomes more comprehensively mapped, SLAM’s success rate gradually surpasses that of RGB-D, reaching a comparable 60% success rate after step 20.

The comparative experiments between the RGB-D and SLAM navigation systems further highlight that the RGB-D system exhibits smoother velocity transitions and smaller velocity deviations, particularly when navigating through complex terrains. During turns, the system shows a controlled deceleration in $v_{x}$ while increasing $v_{y}$, resulting in improved path accuracy and directional adjustments. Moreover, the RGB-D system demonstrates a lower path deviation, with a smaller path length error ($D_{path}$: 0.2797 vs. 0.1777), and it requires fewer iterations for path planning, underscoring its efficiency in dynamically changing environments. Although the SLAM system initially struggles with sparse environmental data, its performance improves as the map becomes more detailed, ultimately achieving a similar success rate of 60% by the conclusion of the experiment.

## 6. Conclusions

In this paper, we successfully ported the MPC- and WBC-based motion control framework from the MIT Cheetah to our dog. This framework has proven effective in providing precise motion control, enabling the robot to navigate complex terrains while maintaining stability. Leveraging this control system, we integrated an RGB-D-based visual navigation approach, resulting in smoother navigation and more efficient path planning. Experiments showed that the RGB-D system achieved a 20% reduction in the cumulative velocity error and a 10% improvement in path tracking accuracy compared to the SLAM-based method, with 15% fewer iterations required for path planning.

Although SLAM was used primarily for comparison, it demonstrated higher computational demands and struggled with navigation in the early stages due to sparse environmental data. Despite this, both systems reached similar success rates by the end of the experiments, with the RGB-D system demonstrating quicker recovery from initial challenges.

The system’s stability is a key area for future improvement, as extended runs in the Ubuntu environment occasionally caused crashes. Another limitation is the reliance on externally provided static maps. Future work will focus on generating global maps in real time, enhancing environmental adaptability and improving real-time path planning. In addition, we plan to explore reinforcement learning-based methods for autonomous navigation, further extending the system’s capacity for real-time decision-making and adaptation in dynamic, unstructured environments. 

## Figures and Tables

**Table 1:** WBC task priority ranking.

Priority	Task Name
0	Optimal reaction force for stance leg
1	CoM posture requirements
2	CoM position requirements
3	Swing leg position

**Table 2:** Trajectory and velocity analysis comparison for RGB-D and SLAM.

Parameter	RGB-D	SLAM
$L_{planned}$: Planned Path Length	8.1084	6.8821
$L_{actual}$: Actual Path Length	8.3881	7.0598
$D_{path}$: Path Length Deviation	0.2797	0.1777
$\sigma_{deviation}$: Std. Dev. of Path Deviations	2.3793	1.7428
$E_{cumulative}$: Cumulative Error (Path)	62.2440	71.1680
$\sigma_{v}$: Std. Dev. of Velocity Deviations	0.0750	0.0771
$E_{v}$: Cumulative Velocity Error	3.8958	6.1356

## Data Availability

No new data were created or analyzed in this study. Data sharing is not applicable to this article.

## Abbreviations

The following abbreviations are used in this manuscript:

COTS	commercial off the shelf
ROS	Robot Operating System
RGB-D	Red–Green–Blue–Depth
SLAM	simultaneous localization and mapping
EKF	Extended Kalman Filter
IMU	inertial measurement unit
ICP	Iterative Closest Point
COM	center of mass
MPC	Model Predictive Control

WBC	Whole-Body Control
PD	Proportional-Derivative
QP	quadratic programming
